# Genetic Relationship, SPAD Reading, and Soluble Sugar Content as Indices for Evaluating the Graft Compatibility of Citrus Interstocks

**DOI:** 10.3390/biology11111639

**Published:** 2022-11-09

**Authors:** Tie Wang, Lijun Deng, Shengjia Huang, Bo Xiong, Muhammad Ihtisham, Zhendong Zheng, Wei Zheng, Zeyu Qin, Mingfei Zhang, Guochao Sun, Jun Wang, Zhihui Wang

**Affiliations:** 1 College of Horticulture, Sichuan Agricultural University, Chengdu 611130, China; 2 College of Agriculture, Forestry and Food Engineering, Yibin University, Yibin 644000, China; 3 Institute of Pomology and Olericulture, Sichuan Agricultural University, Chengdu 611130, China

**Keywords:** citrus, interstock, graft compatibility, antioxidant enzyme, osmoregulation

## Abstract

**Simple Summary:**

Grafting is a critical agricultural practice in citrus growing. The effectiveness of grafting not only depends on the technique but also on the stock–scion combinations. In this study, we investigated the grafting compatibility of five interstock combinations based on physiological and biochemical traits. The results revealed that the grafting compatibility in the early stages of grafting mediated by interstocks was related to the genetic relationship. The leaf chlorophyll content (SPAD reading, soil plant analysis development) and soluble sugar could be employed as preselected indicators to assess compatibility in the late stage of grafting. Our findings lay the foundation for the further research on rootstock–scion interaction mechanism.

**Abstract:**

The interstock, a stock between the rootstock and scion, has a significant regulatory effect on the stock and scion, and its function is highly dependent on graft compatibility. To assess the graft compatibility of the interstock and scion, ‘Yuanxiaochun’ was top grafted onto ‘Ponkan’, ‘Shiranuhi’, ‘Harumi’, ‘Tarocco’, and ‘Kumquat’. The results showed significant differences in the survival ratio and preservation ratio among different combinations. Grafting compatibility in the early stages of grafting was associated with the genetic relationship. The biomass accumulation revealed that the interstock could influence both the rootstock and the scion. The physiological and biochemical traits analysis suggested that SPAD reading and soluble sugar could be employed as preselected indices to evaluate graft compatibility in the late stage of grafting. These results indicated that the evaluation of graft compatibility was a dynamic process. The findings provided a new approach for studying the stock and scion interaction mechanisms mediated by interstock, and directly provided a theoretical and practical basis for the high-grafting of ‘Yuanxiaochun’ citrus.

## 1. Introduction

Grafting, an asexual propagation technique used in agriculture for more than 2500 years, can effectively improve the propagation, production, and multiple stress tolerance of plants [1,2,3]. Citrus, the world’s largest fruit crop group by cultivation area and yield, is mainly produced by grafting techniques [4,5]. Grafting affects the growth [6], flowering [7], and fruit yield and quality [8] of plants. In recent years, with the rapid development of the citrus industry, the speed of variety replacement has accelerated. High-grafting technology with the advantages of rapid variety renewal, quick canopy formation, and easy yield recovery has been widely applied in China [6,9]. However, after high-grafting, a new stock (interstock) was introduced, and the multilevel stock had different effects on the scion [6]. Among these effects, grafting compatibility is the most important one. For example, He et al. [10] reported that ‘Hongmian miyou’ grafted on *‘Poncirus trifoliata’* showed delayed incompatibility. Grafting compatibility between the interstock and scion of citrus has become a limiting factor for high-grafting. Therefore, it is particularly important to select suitable stock and scion combinations.

The compatibility mechanisms of stock and scion grafting have become one of the research hotspots. Previous studies have found that stock–scion combinations exhibit an incompatibility phenomenon in grafting, because the combinations have a certain degree of exclusivity [11], and the fusion cells produced excessive oxidase to affect the grafting survival. In order to understand the mechanism of grafting incompatibility, Mosse [12] described two types of graft incompatibility, ‘translocated’ and ‘localized’. The ‘translocated’ type was characterized by visual observation (etiolating of leaves, which later became redder or more orange, premature defoliation, and leaf wilting). In contrast, anatomical anomalies at the graft union interface, vascular tissue abnormalities in the callus bridge, and bark rupture or discontinuity of the bark–xylem connection were the hallmarks of ‘localized’ incompatibility.

Additionally, genetic relationship, osmotic regulatory substance, antioxidant enzyme and chlorophyll content also play an essential role in the graft compatibility [13,14,15,16]. The genetic relationship is an influential factor affecting the compatibility of grafting. The closer the genetic relationships, the better the grafting compatibility [17]. Among the osmoregulatory substances, soluble sugar is the main form of temporary storage and metabolism of plant energy substances. Li et al. [18] have indicated that exogenous sugar treatment enhanced the xylem reassociation and growth of grafted plants, which further promotes promoted the survival of grafted seedlings. Yeoman et al. [19] found new proteins in tomato autologous stem segments 3 days after grafting, which is hypothesized to be a cellular recognition phenomenon during plant graft formation. Previous studies have shown that grafting directly affects the antioxidant enzyme system of grafted plants [20]. In response to the imbalance of the reactive oxygen metabolism caused by grafting, antioxidant enzymes such as superoxide dismutase (SOD), peroxidase (POD) and catalase (CAT) play corresponding roles [21]. The main role of SOD is to rapidly disproportionate superoxide anion radicals into H_2_O_2_ and O_2_. The POD with CAT breaks down H_2_O_2_ into water. The accumulation of malonaldehyde (MDA) is a sign that the plant is suffering from free radical poisoning [22,23]. The above studies suggest that osmoregulatory substances and antioxidant enzymes are involved in the graft healing process. These studies on stock–scion compatibility focused on the anatomy, physiological and biochemical indicators, and information exchange of the graft interface, which destroyed the plant and prevented continuous observation of samples [24,25,26]. Therefore, attention was turned to the physiological and biochemical indicators of the grafted leaves. The aim was to establish a link between this index and grafting compatibility. For example, Moing et al. [14] found that the soluble sugar and starch contents of leaves were increased in the graft incompatibility combinations at 55 DAG.

The SPAD reading represents the level of chlorophyll content of leaves [27]. A low SPAD reading may be associated with the blockage of carbohydrate assimilates and nitrogen uptake in scion leaves [28]. In addition, some genes are directly implicated in junction formation. For example, *WOX4* (protein-coding genes, a WUSCHEL-related homeobox gene family member with 65 amino acids in its homeodomain) is a potential regulator of graft compatibility, regulating vascular reconnection [26]. Although graft compatibility has been studied deeply and comprehensively on the physiological [29], biochemical, and even molecular levels [30,31], the combined graft compatibility of the stock–scion mediated by citrus interstock is still rarely reported.

In the present study, we selected ‘*Trifoliate orange*’ (*Poncirus trifoliata* (L.) Raf.) as the rootstock, which is the most used in citrus cultivation in China, with high cold resistance and wide adaptability [1]. Five citrus varieties with clear genetic relationships, ‘Ponkan’ (*Citrus reticulata* Blanco cv. Ponkan), ‘Shiranuhi’ [(*Citrus unshiu* × *Citrus sinensis*) × *Citrus reticulata*], ‘Harumi’ [*Citrus reticulata* × (*Citrus reticulata* × *Citrus sinensis*)], ‘Tarocco’ (*Citrus sinensis* L. Osbeck) and ‘Kumquat’ (*Fortunella margarita* Lour. Swingle), were widely cultivated or about to be replaced as interstocks. For example, the cultivation area of ‘Harumi’ has exceeded 130,000 hectares. Sichuan Province is the most suitable ecological zone for late-maturing hybrid citrus in China [6]. ‘Yuanxiaochun’ [(*Citrus unshiu* Marcov × *Citrus sinensis* Osbeck) × (*Citrus reticulata* × *Citrus paradisi*)], a new hybrid citrus variety selected and bred in recent years, has shown good performance in Sichuan Province. To achieve the efficient performance of ‘Yuanxiaochun’ citrus, ‘Yuanxiaochun’ citrus was grafted onto five interstocks, with the aim of understanding the effects of different interstocks on the compatibility of scion grafting. After grafting, we determined the survival and preservation ratio, nutritional development, antioxidant enzyme activities and osmoregulatory substance content in the leaves for each combination. The graft compatibility of the stock and scion combinations was also thoroughly assessed using a mix of observational and anatomical approaches. This study provided an important reference for the interstock graft compatibility and guided high-grafting of ‘Yuanxiaochun’.

## 2. Materials and Methods

### 2.1. Plant Materials and Experimental Design

In March 2018, ‘Ponkan’, ‘Shiranuhi’, ‘Harumi’, ‘Tarocco’ and ‘Kumquat’ scions were grafted onto ‘*Trifoliate orange*’. The grafting height was fixed at 5 cm, the stem diameter was 0.6–0.8 cm, and each cultivar was grafted with 150 plants. Plant spacing was 30 × 30 cm after grafting for colonization and unified management. These plants were employed as an interstock material after survival.

On 15 March 2019, 100 grafted seedlings of ‘Ponkan’, ‘Shiranuhi’, ‘Harumi’, ‘Tarocco’, and ‘Kumquat’ were chosen for unified planting, and the separation distance between varieties was 0.5 m. In the same varieties, the plant spacing and row spacing was 30 × 30 cm, and the diameter of each plant’s graft union was between 0.6 and 0.8 cm. The interstock materials were grafted with ‘Yuanxiaochun’ 30 days after plantation, with a grafting height (interstock length) of 10 cm. At 60 days after grafting (DAG), 60 plants of each interstock variety that survived grafting and grew almost identically were chosen as the test materials, with 20 plants per plot, for three replicates. At 60–180 DAG, the survival and preservation ratios of grafting, determination of vegetative growth, enzyme activities and osmotic regulatory substances in leaves, and graft compatibility were evaluated. All seedlings were handled according to standard procedures in Zhuangjia village, Hongyuan Township, and Jianyang City, Sichuan Province, China.

### 2.2. Statistics on Genetic Relationship Map Construction, Grafting Survival Ratio, and Preservation Ratio

**Figure 1:**
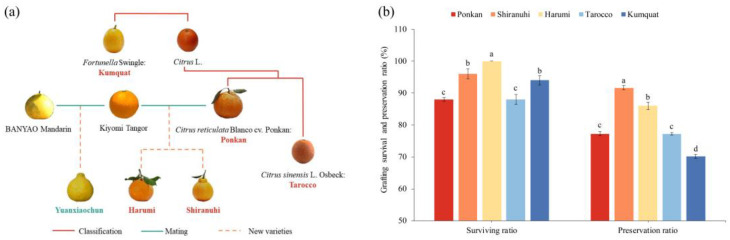
The genetic relationship, graft survival and preservation ratio. (**a**) Profile of the genetic relationship of ‘Yuanxiaochun’ and the five interstocks. The scion font was red, while the interstock materials font was green. (**b**) The effect of different interstocks on grafting compatibility of ‘Yuanxiaochun’ citrus. The data were analyzed using Duncan’s multiple range test in SPSS software. The lowercase letters indicate statistically significant differences among treatments (*p* < 0.05), and data are the mean ± SE of three replicates.

Referring to previous research and the genetic relationship of materials, a genetic relationship map (Figure 1a) was created [32]. The percentage of viable grafted seedlings was determined at 60 DAG (survival ratio), and the same procedure was followed at 180 DAG (preservation ratio) (Figure 1b). These ratios were computed using the formula below: survival ratio = (number of surviving plants at 60 DAG/grafted plants), values are shown in percentages (%); preservation ratio = (number of surviving plants at 180 DAG/number of surviving plants at 60 DAG), values are shown in percentages (%).

### 2.3. Determination of Vegetative Growth and Chlorophyll Content in Leaves

The rootstock, interstock, scion, and lower graft union (graft union of the rootstock and interstock)/upper graft union (graft union of the interstock and scion) diameters were measured with vernier calipers at 240 DAG. In addition, the morphological characteristics of appearance and leaf quality of ‘Yuan Xiaochun’ were counted according to Najla Safaa et al. [33]. The chlorophyll content of the leaves was evaluated using SPAD-502 plus (Konica Minolta, Chiyoda, Japan).

### 2.4. Determination of Enzyme Activities and Osmotic Regulatory Substances in Leaves

Mature functional leaves were collected from each treatment every 30 days from 60 DAG to 240 DAG. The leaf veins were removed and stored in an ultra-low temperature refrigerator at −80 °C until use.

SOD and POD activities were evaluated according to Liao et al. [34], and CAT activity was evaluated by ultraviolet absorption [35]. In addition, the soluble protein, soluble sugar, and MDA contents were measured using the methods of Bian et al. [36], and the free proline content was evaluated using the method of Bates et al. [37].

### 2.5. Evaluation of ‘Translocated’ Incompatibility and ‘Localized’ Incompatibility

At 240 DAG, the ‘Translocated’ incompatibility was categorized according to Moreno et al. [38], whereas ‘Localized’ incompatibility was categorized according to Mosse and Herrero [39] (Table 1).

### 2.6. Statistical Analysis

The data were analyzed using Duncan’s multiple range test in SPSS software (ver. 22.0) at the *p* < 0.05 level of significance.

## 3. Results

### 3.1. Genetic Relationship, Survival Ratio, and Preservation Ratio of Grafting

In terms of genetic relationship, ‘Yuanxiaochun’ was the most closely related to ‘Harumi’ and ‘Shiranuhi’, while ‘Kumquat’ was the most distant (Figure 1a). Interstock treatments affected the survival and preservation ratios of ‘Yuanxiaochun’ (Figure 1b). The survival ratio of ‘Yuanxiaochun’ with ‘Harumi’ as the interstock was much greater, while it was significantly lower for the plants with ‘Ponkan’ and ‘Tarocco’ as interstocks.

The preservation and survival ratios showed different trends. The highest preservation ratio was recorded in the grafting combination with ‘Shiranuhi’ as the interstock, while the lowest was observed in the combination with ‘Kumquat’ as the interstock. The ‘Kumquat’ treatment exhibited the most significant difference in preservation and survival ratio. When the data were combined with a genetic relationship diagram of variations, it was discovered that the closer the genetic relationship with ‘Yunaixiaochun’, the higher the survival and preservation ratios. These findings suggested a relationship between graft compatibility and the genetic relationship in the studied cultivars.

### 3.2. Vegetative Growth

Rootstock, interstock, scion diameters, and the lower and upper graft union diameters all responded significantly to the interaction between stock and scion at 240 DAG (Table 2). Significantly lower vegetative growth of rootstock and scion was observed in ‘Yuanxiaochun’ with ‘Shiranuhi’ as interstock, while the highest vegetative growth level was observed in ‘Yuanxiaochun’ with ‘Kumquat’ as interstock. When compared the combined diameters of different stocks and scions, only the scion/interstock diameter value of the ‘Ponkan’ treatment was larger than 1 (1.13), indicating that this group had an incompatibility phenomenon (scion diameter > stock diameter).

The exterior morphological traits and leaf quality of ‘Yuanxiaochun’ were affected by different interstocks (Table 3). The values of leaf length and leaf shape index of ‘Yuanxiaochun’ with ‘Tarocco’ as interstock were higher, while the ‘Yuanxiaochun’ with ‘Kumquat’ as interstock had a higher fresh and dry mass of 100 leaves. The ‘Yuanxiaochun’ with ‘Harumi’ as interstock had higher dry/fresh quality (the minimum water content). The SPAD reading of ‘Yuanxiaochun’ with ‘Tarocco’ and ‘Kumquat’ as interstock were 80.03 and 76.12, respectively, and that of ‘Ponkan’ treatment was significantly lower (61.87).

### 3.3. Correlation Analysis of SPAD Reading and Vegetative Growth

The relationships between SPAD reading and trunk diameter were investigated in this study. They were significantly positively correlated with interstock diameter and upper graft union diameter (Table 4). In addition, there was a significant correlation between rootstock diameter and interstock diameter.

### 3.4. Antioxidant Enzyme Activities

**Figure 2:**
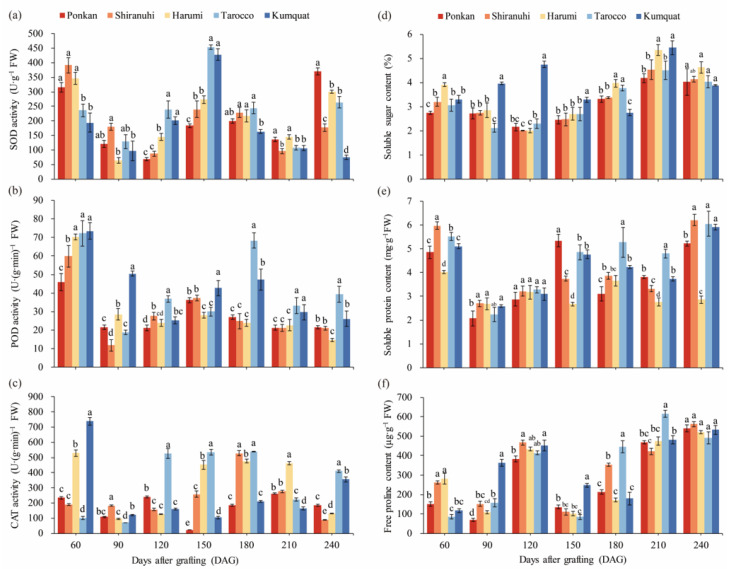
Effects of different interstocks on antioxidant enzyme activities and osmotic adjustment substances of ‘Yuanxiaochun’ citrus leaves. (**a**) SOD, (**b**) POD, (**c**) CAT, (**d**) soluble sugar, (**e**) soluble protein, (**f**) free proline. The data were analyzed using Duncan’s multiple range test in SPSS software. The lowercase letters indicate statistically significant differences among treatments (*p* < 0.05), and data are the mean ± SE of three replicates.

Antioxidant enzyme activities demonstrated a tendency of ‘decrease-increase-decrease-finally climb’ at 60–240 DAG (Figure 2a–c). SOD activity exhibited a definite W-type change trend. POD activity reached its highest point at 60 DAG and the lowest point at 90 DAG. CAT activity of ‘Yuanxiaochun’ with ‘Tarocco’ as interstock was significantly lower at 60 DAG. In conclusion, the antioxidant enzyme activities of each treatment were higher at 60 DAG, and then gradually decreased.

### 3.5. Osmotic Regulatory Substances and MDA Content

Figure 2d–f showed the changes in soluble sugar, soluble protein, and free proline content of scion leaves. ‘Yuanxiaochun’ with ‘Harumi’ as the interstock had a higher soluble sugar content at 60 DAG and 180–240 DAG, while soluble protein content was lower. In contrast, the ‘Yuanxiaochun’ with ‘Tarocco’ as the interstock had lower soluble sugar content but significantly higher soluble protein content. The pattern of ‘rise-descend-rise’ was observed in the free proline content of various treatments. ‘Yuanxiaochun’ with ‘Tarocco’ as interstock had lower free proline content at 60–150 DAG and significantly higher at 180–210 DAG.

**Figure 3:**
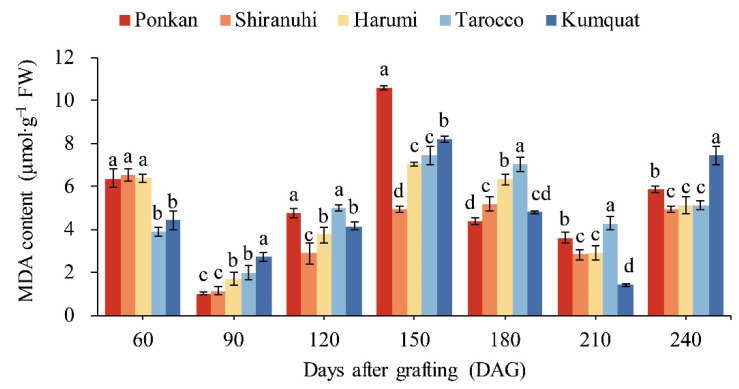
Effects of different interstocks on MDA content of ‘Yuanxiaochun’ citrus leaves. The data were analyzed using Duncan’s multiple range test in SPSS software. The lowercase letters indicate statistically significant differences among treatments (*p* < 0.05), and the data are the mean ± SE of three replicates.

The variations in the MDA content of various treatments were shown in Figure 3, consistent with the overall SOD activity change pattern, which has shown a ‘down-up-down-up’ trend. At 60 DAG, the MDA content of ‘Yuanxiaochun’ with ‘Ponkan’, ‘Shiranuhi’ and ‘Harumi’ as the interstock was higher. Except for the ‘Shiranuhi’ treatment, all treatments had the lowest MDA content at 90 DAG and the highest MDA content at 150 DAG.

### 3.6. Translocated’ Incompatibility and ‘Localized’ Incompatibility

Only ‘Yuanxiaochun’ with ‘Harumi’ as interstock displayed ‘Translocated’ incompatibility signs (Table 5). The ‘Yuanxiaochun’ with ‘Shiranuhi’ as interstock was the only one with a class D, while the ‘Harumi’ interstock treatment received a class B. ‘Ponkan’ and ‘Kumquat’ treatment were consistent, and ‘Tarocco’ treatment had the highest classification evaluation. The treatment compatibility of ‘Tarocco’ as interstock was superior.

## 4. Discussion

### 4.1. Response of Grafting Survival Ratio and Preservation Ratio to Interstocks

As an ancient method of asexual propagation, grafting is widely used for plant propagation, increasing the yield or resistance of horticultural crops [1]. Grafting compatibility has become a research hotspot in botany and the life sciences [26]. The survival ratio and preservation ratio of grafting, as intuitive indicators in the evaluation of graft compatibility, have the advantages of simplicity and easy evaluation. Previous studies reported that there were significant differences in the grafting survival ratio and preservation ratio of various stock–scion combinations [40,41]. In the present study, ‘Harumi’ treatment had a greater survival ratio (100.00%), while ‘Yuanxiaochun’ with ‘Shiranuhi’ as interstock had a higher preservation ratio (Figure 1b).

Grafting is a complex process, in which the genetic relationship is the most important influencing factor [42]. Previous studies have found that intra-species grafting was more compatible than inter-species grafting. That is, graft combinations with similar genetic backgrounds had higher graft compatibility [13,43], which was consistent with our findings. In the present study, we found that, with the exception of the ‘Kumquat’ treatment, the closer the genetic relationship, the higher the survival ratio of grafting at 60 DAG. Additionally, ‘translocated’ incompatibility was observed in some combinations at 240 DAG, including the combinations of ‘Yuanxiaochun’ with ‘Harumi’ as interstock (Table 5). Our results suggested that genetic background might play a more important role in the pre-grafting stage, while the level of grafting compatibility in the later stages of grafting was related to the specific stock–scion combinations.

### 4.2. Interstocks Affect the Accumulation of Scion Biomass

Stock–scion interactions have been shown to affect scion biomass accumulation in apples and *Prunus dulcis* [44,45], which was consistent with our findings. In this study, ‘Yuanxiaochun’ with ‘Shiranuhi’ and ‘Harumi’ as interstock had lower vegetative growth levels in the rootstock, interstock, and scion (Table 2). Compared with other stock–scion combinations, a higher scion/interstock diameter was recorded in the ‘Ponkan’ treatment (>1), which indicated that the combination had a mild incompatibility phenomenon. Based on the results of previous studies, this might be due to the inconsistent growth rate of rootstock and scion or blockage of substances transport [15,46].

Leaf characteristics affect the photosynthetic capacity of plants and further affect the yield [47]. The dry mass of leaves reflects the ability of the scion to accumulate nutrient elements. According to our study, ‘Yuanxiaochun’ with ‘Kumquat’ as interstock had a higher level of the dry mass of leaves, indicating that the plant invested a higher percentage of leaves and showed a strong tolerance (Table 3). Similar results have been demonstrated in oranges and plums [48,49]. The lower SPAD reading may indicate that carbohydrate and nitrogen uptake in the scion leaves is hindered [28]. Moreno et al. [50] reported that scion might influence carbon and nitrogen assimilation in rootstock via the phloem, which often occurs in the first year after grafting [51]. As a result, the SPAD reading and trunk growth status are employed to assess graft compatibility [15]. In this study, we also found that the diameter of the interstock and the graft union all positively correlate with the SPAD value (Table 4), indicating that the SPAD reading could be used as a preselection index for the evaluation of the graft compatibility of stock and scion.

### 4.3. Response of Antioxidant Enzymes and Osmoregulatory Substances to Graft Compatibility

The physiological and biochemical indicators could reflect the graft compatibility [52,53]. The antioxidant enzymes, such as SOD, POD, CAT, and other reactive oxygen-scavenging systems in plants as plant defense systems are able to defend against adverse external conditions and mitigate damage to the plant [54]. In the present study, SOD, POD and CAT activities were all higher at 60 DAG (Figure 2a–c), and it was reported that this might be related to the incomplete healing of the interstock and scion in grafting. The antioxidant enzyme activities were significantly higher in the treatment with ‘Harumi’ as the interstock at 60 DAG, which was consistent with a higher grafting survival ratio, and similar conclusions were reached by Chen et al. [24]. However, the overall lower antioxidant enzyme activities and significant ‘Translocated’ incompatibility symptoms were observed in ‘Yuanxiaochun’ with ‘Harumi’ as interstock at 240 DAG. In contrast, ‘Yuanxiaochun’ with ‘Tarocco’ as interstock with better grafting compatibility evaluation and higher antioxidant enzyme activities. This implied that graft compatibility was related to antioxidant enzyme activities, and graft compatibility assessment was a dynamic and complex process. In addition, SOD and POD activities showed similar trends in the grafted combinations leaves, while CAT activity showed stable performance. This result indicated that the functions and contributions of various protective enzymes differed at different periods of plant grafting.

Soluble sugar plays an important role in the evaluation of grafting compatibility. Previous studies have found that sugar promotes graft union development in the heterograft of cucumber onto pumpkin [18]. However, the accumulation of soluble sugar in the leaves of grafted plants is associated with grafting incompatibility, and rootstocks in incompatible combinations induced soluble sugar enrichment in the leaves of the scion [14]. In our study, similar results were demonstrated. The treatment with ‘Harumi’ as interstock had a significantly higher soluble sugar content at 60, 180–240 DAG, and a significant decrease in survival ratio (Figure 1b). In addition, the grafted plants exhibited smaller scion diameter, leaf length and width, and ‘translocated’ incompatibility symptoms (Table 2 and Table 3 and Figure 2d). This might be accounted for by the altered sugar transport brought on by grafting incompatibility [55].

In addition, the soluble proteins are important for the early growth stages of linkage between rootstock combinations, and facilitate the formation of initial adhesions between rootstocks [56,57]. It also has a very important role in the leaves of grafted plants and could be used as a pre-selection indicator for grafting compatibility evaluation. Xu et al. [58] found that the graft–compatibility combination had higher levels of protein expression along with superior physiological and growth characteristics. This can be explained by the higher expression of proteins involved in photosynthesis, carbohydrate and energy metabolism, and protein metabolism. In this study, the soluble protein content was higher at 60 DAG, which further confirmed the response of grafting. Among them, ‘Yuanxiaochun’ with ‘Harumi’ as interstock had significantly lower soluble protein levels at 60, 150–240 DAG (Figure 2e) and poorer growth characteristics (Table 2 and Table 3). It was further found that the free proline and MDA content of ‘Yuanxiaochun’ with ‘Harumi’ as interstock were higher at 60 DAG. It is well known that the accumulation of free proline and MDA plays a key role in the plant response to stress [59,60]. The above results indicated that the grafting compatibility of ‘Yuanxiaochun’ with ‘Harumi’ as interstock at 60 DAG might have started to deteriorate.

Among these indicators, antioxidant enzymes showed different dynamic trends with grafting time, which was not favorable for the evaluation of grafting compatibility. In contrast, soluble sugar showed better agreement with grafting compatibility, and we suggest that soluble sugar in the scion leaves might be one of the preselected indicators of grafting compatibility.

## 5. Conclusions

The evaluation of grafting compatibility is a long and complex process. This study revealed that grafting compatibility was associated with genetic relationship, SPAD reading, and soluble sugar content of leaves. The closer the genetic relationship, the higher the survival ratio of grafting in the early stages of grafting. In the late stages of grafting, the grafted combinations with lower SPAD reading and higher soluble sugar content of leaves displayed worse compatibility and growth characteristics. Our experimental results should be further understood by setting up more controlled experiments, increasing the variety sample and continuing to extend the evaluation time. Overall, ‘Tarocco’ and ‘Kumquat’ were the most suitable as interstocks for ‘Yuanxiaochun’.

## Figures and Tables

**Table 1:** ‘Translocated’ incompatibility and ‘Localized’ incompatibility.

‘Translocated’ Incompatibility Symptoms	‘Localized’ Incompatibility Category
Leaf and wood yellowing and reddening, defoliation, tree vigor reduction, and death. Moreover, the tree body may have low SPAD values.	Category A: Perfect unions (the line of the union between bark and wood is hardly visible); Category B: Good unions (the bark and wood are continuous although the line of a union in the wood is often clearly distinguished by excessive ray formation); Category C: Unions with discontinuities in the bark (the bark tissues of rootstock and scion are separated by a dark brown layer of corky appearance); Category D: Unions showing vascular and wood discontinuities (the woody tissues of rootstock and scion are separated in many places by clusters of living, non-lignified parenchyma, whereas bark tissues are generally as in Category C); Category E: Observed breakage of the tree at the graft union in the nursery or orchard. Categories D and E were considered ‘incompatible’ unions because breakage might occur caused by mechanical damage or wind.

**Table 2:** Effects of different interstocks on vegetative growth.

Treatment	Rootstock Diameter /mm	Interstock Diameter /mm	Scion Diameter /mm	Lower Graft Union Diameter /mm	Upper Graft Union Diameter /mm	Interstock/Rootstock Diameter	Scion/Interstock Diameter	Upper/Lower Graft Union Diameter
Ponkan	17.08 ± 0.61 a	8.73 ± 0.16 bc	9.83 ± 0.44 a	13.24 ± 1.13 bc	11.32 ± 0.63 bc	0.51 ± 0.05 c	1.13 ± 0.06 a	0.86 ± 0.04 ab
Shiranuhi	11.97 ± 0.70 b	7.60 ± 0.10 c	5.91 ± 0.18 c	12.58 ± 0.19 c	11.07 ± 0.17 c	0.64 ± 0.03 ab	0.78 ± 0.01 b	0.88 ± 0.02 ab
Harumi	16.77 ± 1.01 a	8.43 ± 0.63 bc	7.51 ± 0.27 b	15.67 ± 0.82 b	10.22 ± 0.85 c	0.50 ± 0.01 c	0.90 ± 0.08 b	0.66 ± 0.08 b
Tarocco	18.00 ± 1.56 a	10.18 ± 1.04 ab	8.94 ± 0.18 a	15.67 ± 1.26 b	14.49 ± 0.48 ab	0.56 ± 0.02 bc	0.90 ± 0.11 b	0.93 ± 0.07 a
Kumquat	17.26 ± 1.15 a	11.42 ± 0.52 a	10.05 ± 0.66 a	18.94 ± 0.70 a	15.37 ± 1.95 a	0.66 ± 0.02 a	0.88 ± 0.03 b	0.82 ± 0.14 ab

The data were analyzed using Duncan’s multiple range test in SPSS software. The lowercase letters indicate statistically significant differences among treatments (*p* < 0.05), and data are the mean ± SE of three replicates.

**Table 3:** Effects of different interstocks on the external morphological characteristics and leaf quality of ‘Yuanxiaochun’.

Treatment	Leaf Length /cm	Leaf Width /cm	Leaf Shape Index	Fresh Mass of 100 Leaves /g	Dry Mass of 100 Leaves /g	Dry/Fresh Quality	Water Content /%	SPAD Reading
Ponkan	8.72 ± 1.04 bc	3.33 ± 0.35 ab	2.66 ± 0.24 b	26.84 ± 0.48 c	10.86 ± 0.09 c	0.40 ± 0.00 bc	59.52 ± 0.40 ab	61.87 ± 1.85 c
Shiranuhi	9.55 ± 0.28 ab	3.07 ± 0.26 ab	3.16 ± 0.29 ab	28.96 ± 0.48 b	11.21 ± 0.05 c	0.39 ± 0.00 c	61.26 ± 0.82 a	70.02 ± 2.07 b
Harumi	8.00 ± 0.46 c	2.70 ± 0.24 b	2.98 ± 0.12 ab	29.86 ± 0.40 b	13.29 ± 0.10 b	0.45 ± 0.00 a	55.49 ± 0.28 c	66.42 ± 2.14 bc
Tarocco	10.53 ± 0.30 a	3.02 ± 0.11 b	3.50 ± 0.07 a	26.80 ± 0.51 c	10.43 ± 0.02 d	0.39 ± 0.00 bc	61.04 ± 0.67 ab	80.03 ± 0.03 a
Kumquat	9.80 ± 0.56 ab	3.80 ± 0.10 a	2.58 ± 0.09 b	35.68 ± 0.39 a	14.50 ± 0.27 a	0.41 ± 0.00 b	59.36 ± 0.31 b	76.12 ± 1.33 a

The data were analyzed using Duncan’s multiple range test in SPSS software. The lowercase letters indicate statistically significant differences among treatments (*p* < 0.05), and data are the mean ± SE of three replicates.

**Table 4:** Correlation analysis of SPAD reading and vegetative growth.

	Rootstock Diameter	Interstock Diameter	Scion Diameter	Lower Graft Union Diameter	Upper Graft Union Diameter	SPAD Reading
Rootstock diameter	1					
Interstock diameter	0.709 **	1				
Scion diameter	0.663 **	0.649 **	1			
Lower graft union diameter	0.402	0.651 **	0.426	1		
Upper graft union diameter	0.552 *	0.836 **	0.577 *	0.455	1	
SPAD reading	0.289	0.554 *	0.133	0.379	0.733 **	1

**, At the 0.01 level (two-tailed), the correlation is significant; *, At the 0.05 level (two-tailed), the correlation is significant.

**Table 5:** Effects of different interstocks on the graft union of ‘Yuanxiaochun’.

Treatment	‘Translocated’ Incompatibility Symptoms	‘Localized’ Incompatibility Category
Ponkan	N	1A, 5B
Shiranuhi	N	4B, 2D
Harumi	Ab	6B
Tarocco	N	2A, 4B
Kumquat	N	1A, 5B

Abbreviations: N, visual normal trees; Ab, abnormal scion behavior, reduction in vigor, leaf yellowing (refer to Table 1).

## Data Availability

Not applicable.
